# Communication needs for improved uptake of PrEP and HIVST services among key populations in Nigeria: a mixed-method study

**DOI:** 10.1186/s12981-021-00411-6

**Published:** 2021-11-20

**Authors:** Olawale Durosinmi-Etti, Emmanuel Kelechi Nwala, Funke Oki, Akudo Ikpeazu, Emmanuel Godwin, Paul Umoh, Arome Shaibu, Alex Ogundipe, Abiye Kalaiwo

**Affiliations:** 1 JSI Research and Training Institute Inc, Abuja, Nigeria; 2 National Agency for the Control of AIDS, Abuja, Nigeria; 3 National AIDS and STIs Control Program, Federal Ministry of Health, Abuja, Nigeria; 4 Heartland Alliance, Abuja, Nigeria; 5 USAID, Abuja, Nigeria

## Abstract

**Background:**

World Health Organization (WHO) reports that people who indulge in risky behaviours such as penile-anal sex, unprotected intercourse, multiple sex partners, and alcohol and illicit drugs are at risk of HIV/AIDS and classified as Key Populations (KPs). Since the introduction of PrEP and HIVST for the key population groups in Nigeria, government entities and implementing partners have used a range of channels in messaging these essential services across to the target groups—ranging from in-person, social media, television, and radio adverts. Yet, few successes have been documented, thereby necessitating the need to understand the enabling facilitators, barriers to, and communication needs of the KP groups in messaging PrEP and HIVST services in Nigeria. Communicating PrEP and HIVST services will empower the key populations to seek available HIV prevention services and help to increase access to HIV testing services in Nigeria.

**Methods:**

This study was a mixed-method cross-sectional design; involving 1169 participants from the key populations in Nigeria. The study used a survey and qualitative exploratory methods (interviews and focus group discussion), to collect data from the participants—MSM, FSWs, and key influencers of the KP groups (health providers, peer educators, HIV program officers). In August 2020, data collection was conducted using an open data kit (ODK). Quantitative data were analyzed using SPSS version 20 for descriptive statistics, while qualitative data were analyzed using deductive and thematic analysis based on the codebook.

**Results:**

The KPs were mainly urban dwellers (77.7%), and the majority of the participants were between 18 to 28 years (89.3%). However, the MSM group was of a younger population compared to the FSWs. A majority completed secondary education (56.1% FSWs and 43.5% MSM). The MSM group showed more tendency to acquire higher education compared to the FSWs. For example, about 51.3% of the MSM group were undergraduates compared to 9.5% of the FSWs. The majority of the KPs were self-employed (56.4% FSWs and 40% MSM). Only about 51% of the KPs were aware of PrEP, with typological variations (39.9% FSWs and 62.3% MSM). MSM group in Lagos (82.5%) were more aware of PrEP services, than 53.1% and 54.5% in A/Ibom (53.1%) and C/River (54.5%). Among the enablers to acquiring PrEP information was the ability of the KPs to network within their communities and on personal relationships. Evidence shows that no single approach influenced the acquisition and use of PrEP information by KPs. Although this proportion varied across the geographic locations, only about 50% of the KPs were aware of HIVST services (40% FSWs and 60% MSM). The factors that enabled the acquisition and use of the prevention commodities were cross-cutting, including a previous or current role as a peer educator, integration of the messages, peer networking, multi-lingual and multi-channel presentation, job aids, and reminders. KPs expressed the need for information on how to take PrEP, eligibility, clarification on differences between PrEP and PEP, clarification on any side effects, for PrEP, price, efficacy, sales point, dosage, available brands. A scale-up of the research across all geopolitical zones and a survey to quantify the prevalence would help understand the dynamics and prioritization of interventions for scaling up PrEP and HIVST services in Nigeria.

**Conclusions:**

The study documented barriers and facilitators to the uptake of PrEP and HIVST among key populations in Nigeria. It highlighted that KPs are willing to receive PrEP and HIVST messages. The policy actors should consider the preferences of the KPs and the key influencers in reducing barriers to communication and increasing the uptake of PrEP and HIVST services; ensure it reflects in a tailored communication strategy. Since multi-linguistics and multi-channels of presentation were enablers to acquiring PrEP and HIVST messages, the communications strategy for HIV prevention should incorporate these recommendations and adapt to context-specific approaches for effective messaging.

## Introduction

World Health Organization (WHO) reports that people who indulge in risky behaviours such as; penile-anal sex, unprotected intercourse, multiple sex partners, and alcohol and illicit drugs are at risk of HIV/AIDS and classified as Key Populations (KPs) [1–3]. Though there has been progress in reducing death due to AIDS, the number of new HIV infections has been on the increase, especially among the KPs, including men who have sex with men (MSM) and female sex workers (FSWs) [4–6].

There have been multiple interventions to address the burden of HIV [1, 7] as part of the strategy of reaching the UNAID’s 95–95–95 goals – of having 95 of people living with HIV diagnosed, 95% of diagnosed on antiretroviral therapy (ART), and 95% of treated virally suppressed by the year 2030. In Nigeria, despite the introduction of pre-exposure prophylaxis (PrEP) and HIV self-testing (HIVST) services in 2017 and 2018, there is reportedly low uptake of HIV prevention services among the KPs [8, 9]. Factors related to cost, literacy, language, norm, culture, type of sex work, scheduling, individual and group behavioural patterns, risk perception, perceived relevance of interventions, contextual factors, norm, stigma and discrimination, and constant migration among the KP groups, continue to compound low uptake of the HIV prevention services [10–14]. Addressing these barriers requires institutionalizing an effective and well-implemented communication strategy. These include—creating awareness, initiating, sustaining, and maintaining a positive and desirable behaviour to prevent HIV infection among KPs.

Since the introduction of PrEP and HIVST for the key population groups in Nigeria, government entities and implementing partners have used a range of channels in messaging these essential services across to the target groups—ranging from in-person, social media, television, and radio adverts [7, 15, 16]. Yet, few successes have been documented, thereby necessitating the need to understand the enabling facilitators, barriers to, and communication needs of the KP groups in messaging PrEP and HIVST services in Nigeria. For example, a cohort pilot conducted in Lagos using a hotline to disseminate messages HIVST kits among 257 participants showed that 98% of the participants requested access to the HIVST kits due to the health information [7]. Also, a study in northern Nigeria indicated that only about 53% of the participants were aware of PrEP. Participants reported social networking app as a medium for receiving information about PrEP [12]. This knowledge gap necessitated the need to document the enabling facilitators, barriers to, and the needs of the KP groups in communicating PrEP and HIVST services in Nigeria. Messaging PrEP and HIVST services will empower the key populations to seek available HIV prevention services and help to increase access to HIV testing services in Nigeria.

The purpose of the study was to identify the communication needs and preferences of key population (KP) groups as evidence for developing strategies and interventions to increase awareness and use of HIVST and PrEP services among the KPs in Nigeria. Specifically, the study aimed to:

- To identify the enablers in acquiring and using effective communication for promoting PrEP and HIVST among KP groups in Nigeria
- To identify the barriers to acquisition and use of communication required to promote PrEP and HIVST use among KP groups in Nigeria.
- To describe communication needs of KPs and their support groups to promote behavior change for PrEP and HIVST among KP groups in Nigeria.

## Methodology

### Study sites

Akwa Ibom, Cross River, and Lagos states were selected because they are priority states where the Total Market Approach for HIV Prevention Programming in Nigeria Project is implementing. A profile of the study areas is outlined in Table 1 [16–18].

**Table 1:** Profile of study states in Nigeria

Indicators	Akwa Ibom	Cross River	Lagos
HIV prevalence	5.6%	1.7%	1.3%
Population and landmarks	South-south Nigeria, 7081 km^2^, estimated population of 5,540,758	South-south Nigeria, 20,156 km^2^, estimated population of 3,737,517	South-west Nigeria, 3577 km^2^, estimated population of 14,368,332
Median age at first sexual intercourse	17.4 for women, 22.3 for men	17.5 for women, 19.2 for men	20 years for women, 20.3 years for men
% of women with sexual activity within the past 4 weeks	42.9%	46%	51.2%
% of women who never had sexual intercourse	15.1%	12.1%	20%
% of men with sexual activity within the past 4 weeks	50%	43.5%	57.8%
% of men who never had sexual intercourse	16.8%	18%	13.4%
Fertility rate	3.6	3.7	3.4
% of people with a discriminatory attitude toward people with HIV	61.1% for women, 38.2% for men	28.8% for women, 40.4% for men	66.9% for women, 65.3% for men
% who reported using a condom during last sexual intercourse with such a partner	33% for females, 72.5% for men	37.9% for females, 65.5% for men	38.9% for female, 71.5% for men

### Study design and population

This study was a mixed-method cross-sectional design; involving a survey of 1169 participants; qualitative exploratory methods; focus group discussion (FGD), and key informant interview (KII) among MSM, FSWs, and key influencers of the KP groups (health providers, peer educators, HIV program officers).

### Sample size

The table below describes the different data collection activities for this research. The quantitative sample calculation was based on previous key population characterization in 2015 by Society for Family Health [19], from a known population size—n = $\frac{NN}{1+NN(ee)2}$. Based on the estimated population of the KPs in each state as documented in a previous study [19], the different sample size was calculated for each state (see Table 2). For qualitative interviews, focused group discussions (FGDs), were conducted with the KP groups—MSM and FSWs. Also, key informant interviews (KIIs) were conducted with the KPs and key influencers across the three states. (see Table 2 for breakdown).

**Table 2:** Sample distribution for quantitative and qualitative interviews

	A/Ibom	C/River	Lagos	Total
Questionnaire administration				
FSWs	225	286	313	824
MSM	143	99	103	345
Qualitative interviews				
FGD with MSM	1	1	1	3
FGD with FSWs	1	1	1	3
KIIs with MSM	10	10	10	30
KIIs with FSWs	10	10	10	30
KIIs with KP key influencers	17	17	15	49

### Data collection and management

The survey was conducted through a face-to-face interaction using an open data kit (ODK). In addition, FGDs and KIIs were conducted with a topic guide in August 2020. KPs above 18 years and consented to the study were included; the KPs were recruited through their support groups, led by key influencers. Data were collected on KPs’ socio-demographic characteristics, awareness of PrEP and HIVST, and preferred communication channels for PrEP and HIVST messaging. Interviews were conducted using the respondents' preferred language, recorded, and transcribed verbatim to ensure data quality. The transcripts were given unique identifiers for ease of analysis and reference.

### Data analysis

Quantitative data were analyzed using SPSS version 20 for descriptive statistics, while qualitative data were analyzed using deductive and thematic analysis based on the codebook. The codebook is an analysis framework for qualitative exploratory studies, containing themes and sub-themes for the interpretation of data.

## Results

### Participants characteristics

A total of 1169 (824 FSWs and 345 MSM) KPs participated in the survey. Sixty KIIs were conducted (30 FSWs and 30). We conducted 6 FGDs (3 FSWs and 3 MSM groups). In addition, a total of 49 KIIs were conducted with key influencers (27 males and females). Results indicated that KPs were mainly urban dwellers, and the majority of the participants were between 18 to 28 years. However, the MSM group was of a younger population compared to the FSWs. The MSM group showed more tendency to acquire higher education compared to the FSWs. For example, about 51.3% of the MSM group were undergraduates compared to 9.5% of the FSWs (p = 0.000). The majority of the KPs were self-employed (Table 3).

**Table 3:** Participants’ characteristics

KP group	FSW		MSM	
	(n =)	(%)	(n =)	(%)
Sample size	824	70.5	345	29.5
Age				
18–28 years	526	63.8	308	89.3
29–38years	229	27.8	34	9.9
< 38 years	69	8.4	3	0.9
Place of residence				
Rural	304	36.9	77	23.3
Urban	520	63.1	268	77.7
Level of education				
Primary not completed	60	7.3	0	0
Primary completed	94	11.4	2	0.6
Junior secondary completed	115	14	1	0.3
Senior secondary completed	462	56.1	150	43.5
Advanced level	10	1.6	6	1.7
Undergraduate	78	9.5	177	51.3
Postgraduate	5	0.6	9	2.6
Occupation				
Government employed	3	0.4	9	2.6
Private sector	18	2.2	30	8.7
Self-employed	465	56.4	138	40
Student	52	6.3	137	39.7
Unemployed	210	25.8	57	16.5
Others eg retired	76	9.2	4	1.4

### Awareness about PrEP services

The mixed-method analysis revealed disproportionate gaps in awareness about PrEP among the KPs. Only about 51% of the KPs were aware of PrEP, with typological variations (39.9% FSWs and 62.3% MSM; p = 0.000). The level of awareness was higher among those who previously served as peer educators to the KPs. However, there was some misconception around the eligibility for the use of PrEP—for example, some KPs mistake pre-exposure prophylaxis of HIV (PrEP) for post-exposure prophylaxis (PEP). Such information asymmetry continues to limit the introduction and use of PrEP among the KPs despite understanding the importance of PrEP in preventing HIV.

“I thought it’s when you have unprotected sex or if condom breaks during sexual intercourse, then you can take the drug” LAS-KII-FSW-09.

MSM group in Lagos (82.5%) were more aware of PrEP services, than 53.1% and 54.5% in A/Ibom(53.1%)and C/River (54.5%) (p = 0.000). In addition, the MSM group showed a higher level of awareness of PrEP than the FSW group.

**Fig. 1:**
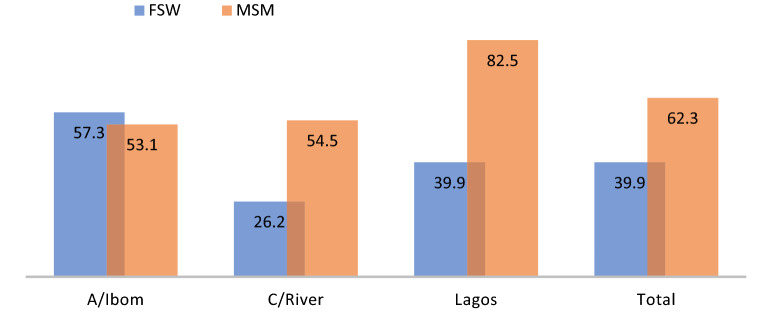
Awareness about PrEP

Data shows multiple, intertwining information flow for PrEP, including peer influence, social media, NGO/CBO activities, health providers, among others. MSM group was likely to acquire PrEP information via peer influence (46%) and social media (14.4%) compared to the FSW group (24.2% and 6.1%), respectively (p = 0.000). Contrarily, FSWs reported higher proportions of NGO/CBO and health provider influence (usually face-to-face messaging) than the MSM group across the three states (see Fig. 1).

Among the enablers to acquiring PrEP information was the ability of the KPs to network within their communities and on personal relationships. Information about PrEP continues to diffuse among the KPs—some community members reported receiving or giving out information on PrEP and HIVST to other community members who do not participate in the peer sessions or are not in the closed community groups. MSM group was more likely to network information on PrEP than FSWs.

### Current channels for messaging PrEP services

**Fig. 2:**
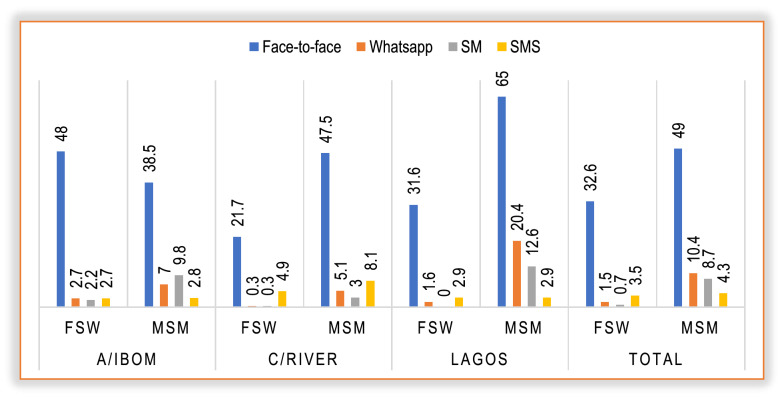
The proportion of reported channel of messaging for PrEP in Nigeria

Evidence shows that no single approach influenced the acquisition and use of PrEP information by KPs. However, the Face-to-face channel was the most reported means of acquiring PrEP messaging (32.6% FSWs and 49% MSM) (see Fig. 2).

Findings showed that KPs explored various media channels for messaging PrEP. This included WhatsApp, Facebook, Instagram, and Twitter, and short message service (SMS). However, using mixed languages—local dialect, pidgin, and English languages for messaging PrEP across the three states enabled acquisition and use of such information. Other channels are exclusively for KPs such as manjam, tindre, and grindr, providing more secure, encrypted opportunities for KPs to express themselves and share information with their key influencers. These channels were yet to be maximized.

“Yes, Instagram, Facebook, we have even some Apps such as manjam, tindre, and grindr that are strictly for community members” LAS-KII-PM-02.

“I get the information from the internet, mass media. There are even groups on WhatsApp, Facebook that you can belong to talk about PrEP” AKS-FGD-MSM-P1.

Complimentarily, key influencers reported using critical job aids to facilitate PrEP and HIVST awareness within KP communities irrespective of typology.

“We go with posters and even fliers, and we show them all those things” LAS-KII-HCP-03

### Awareness about HIVST services

**Fig. 3:**
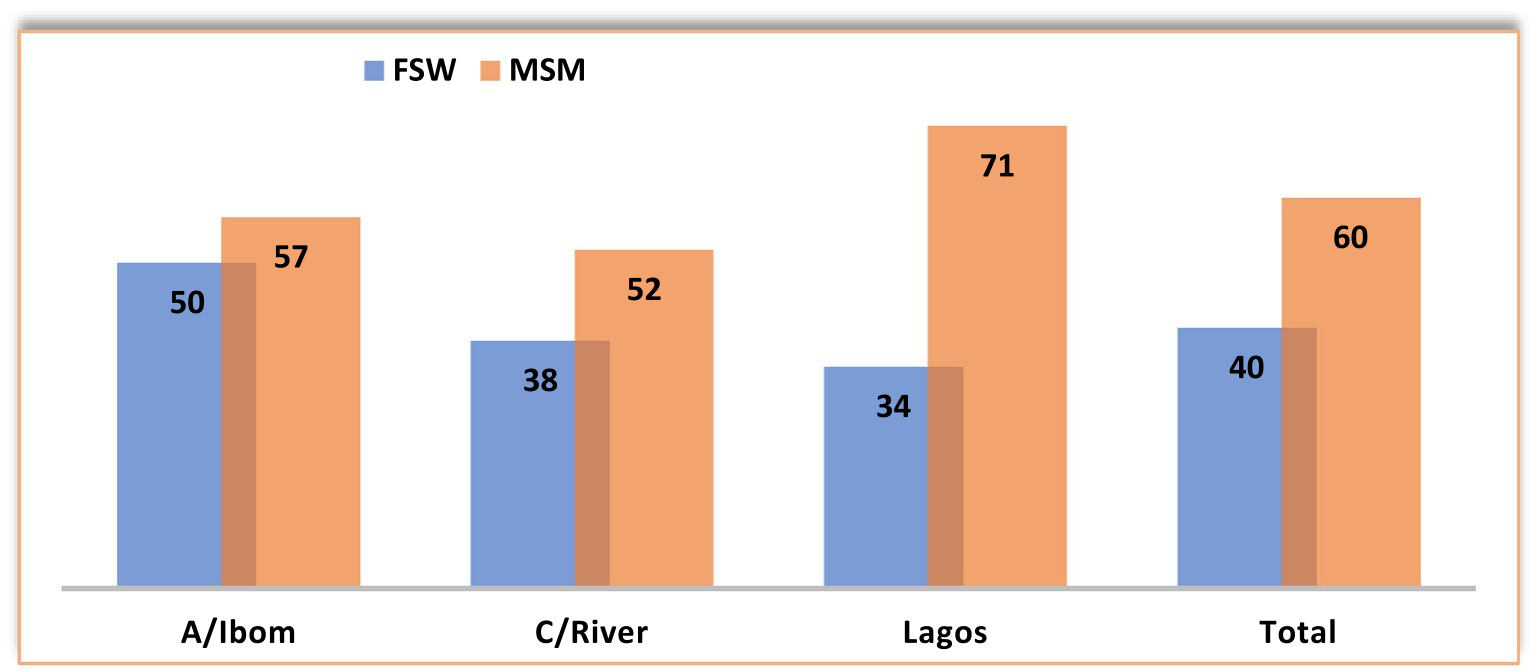
Awareness about HIVST services

Gaps exist in awareness about HIVST, especially among the FSWs. Only about 50% of the KPs were aware of HIVST services (40% FSWs and 60% MSM), although this proportion varied across the geographic locations (p = 0.004). (see Fig. 3).

Mixed reactions from the respondents suggested that even though there has been an effort to increase HIVST awareness, there may have been slow progress in the uptake of the services.

“For now, we are just aware of HIVST, but we have not started using it” LAS-KII-MSM-03.

“I don’t know about it before now, but If I see it, I can use it on myself” LAS-KII-FSW-01.

### Current channels for messaging HIVST services

For HIVST messaging, the in-person channel was the most reported (85% FSWs and 68% MSM). The second was social media channels, including Facebook, Twitter, and Instagram (9.4% FSWs and 31.6% MSM). These proportions varied across the study locations. In line with the COVID-19 social distancing protocol, key influencers reported that the social media channels also act as social support groups which they use to upload videos displaying information and demonstrating how to and how not to conduct self-test for HIV.

**Fig. 4:**
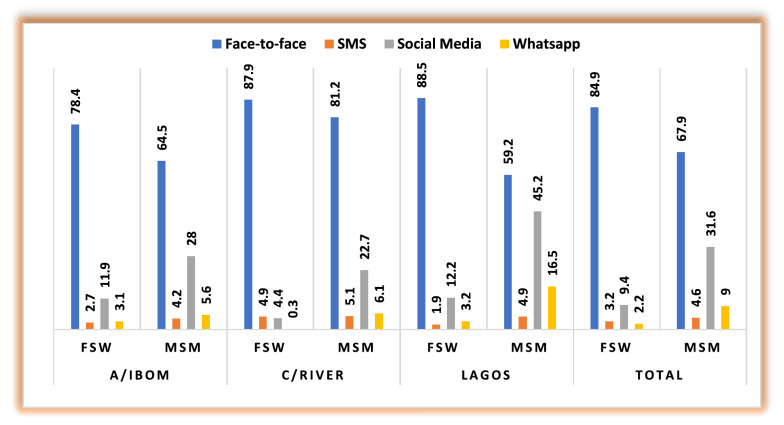
Channel of messaging for HIVST services in Nigeria

FGDs indicated an integrated delivery of HIVST with other commodities such as PrEP, condoms, and lubricants, given that they all target the same population groups (Fig. 4).

Qualitative data indicated that some MSM reported that they became aware of HIVST initially during peer group discussion and subsequently through NGOs, social media, flyers, emails, and face-to-face with a health care provider. While many of the respondents did not have a good understanding of the workability of the self-testing process, there were a selected few who could describe the procedures, despite using some KPs as ambassadors for promoting HIVST services.

“The information I heard is that the HIV self-testing is a kit being used personally at your own convenient time to test yourself either from oral fluids or from blood. “ CRS-KII-MSM-07.

Cross-sectional views of the respondents suggested that various channels encourage wider communication of HIVST information, indicating that there is not one channel for effective delivery of the information.

“We use Facebook, WhatsApp, Instagram, witter” LAS-KII-Key influencer-01.

“I belong to a group on Facebook where I get HIV messages > That is how I got to know of the PrEP we are talking about now” CRS-FGD-MSM.

### Enablers to acquisition and use of PrEP and HIVST messaging among the KPs

The factors that enabled the acquisition and use of the prevention commodities were cross-cutting and outlined in Table 4 below:

**Table 4:** Enablers to Acquisition and Use of PrEP and HIVST Information among the KPs

Enablers	PrEP	HIVST
Participation in peer education sessions/previous or current role as peer educators	X	X
Integration of the messages with other commodities	X	X
Removal of user fees for PrEP	X	□
Community linkages/networking—especially among MSM	X	X
Multi-lingual presentation of the messages	X	X
Use of diverse channels for peer sessions—face-to-face, social media (Facebook, Instagram, and WhatsApp)	X	X
Use of job aids and experiential learning approaches	X	X
Reminders via phone call or SMS outreach program	X	□

X = presence of enabler;  □ = absence of enabler

### Barriers to acquisition and use of PrEP and HIVST messages

Barriers to acquisition and use of PrEP and HIVST messages found in this study are categorized into five typologies: individual, facility-related, socio-cultural, socio-economic, and medication-related factors.

#### Individual factors

There is initial self-stigma due to the level of literacy required to participate in the social media-based peer session, retrieving messages, and navigating through the platforms. This phenomenon is preponderant among FSWs than MSM and leads to KPs pairing with their friends to receive social media/device-based information on the prevention commodities.

“Some people don't know how to read and write - some of them will choose to join their friends to do it because their friends are doing it. Some will feel ashamed and isolate themselves because they can't read” LAS-KII-FSW-07.

“There are people who may not be comfortable with your sexual orientation and these commodities, because of this kind of situation, you can't hide like that. So, you have to tell the truth” AKS-KII-MSM-04.

The absence of privacy limits the acquisition of information on PrEP and HIVST services. Some participants do not have employment and share accommodation and personal effects with other household members. Therefore, they do not have privacy in keeping and using these essential HIV prevention commodities. Some MSM felt that closed groups such as WhatsApp and Facebook are subject to abuse with gossip and intangible discussions, leading to a lack of willingness of some community members to participate in the discussions.

“The reason, I don't subscribe to this WhatsApp of a thing is, in the beginning, it would be interesting, but at the middle and the end, it's all gossips and stuff that make it boring and uninteresting” LAS-FGD-MSM-P3.

Although there were positive responses regarding messaging through social media, some KPs complained that they do not have smartphones required to access social media interactions. Some FSWs complained about losing the social media handle addresses (Instagram and Twitter), while some have never been on social media.

“For now, I am not on any social media platform because I don't have an android phone, because they stole it” LAS-KII-FSW-01.

Reports also indicated that social media-based communication messages could be misinterpreted sometimes, probably due to a lack of understanding of PrEP or HIVST messages. However, KPs recognized that social media saves cost and time to attend face-to-face sessions.

KPs often expect incentives (financial and other non-financial rewards) before participation in information sharing sessions, especially when it involves in-person peer sessions. Non-financial incentives highlighted included the provision of free condoms, lubricants, and HIVST kits. Financial reward relates to transport fees despite that peer educators visit the KP locations, especially for the FSWs.

“You know here in Nigeria if you want to get somebody's mind, there must be something like an incentive to attract them” LAS-KII-Key influencer-03.

Frequent low phone battery (resulting from the limited power supply) and limited internet access were among the potential barriers to effective communication of PrEP and HIVST services among the FSWs since it has to do with social media platforms. For those KPs who participate in the social media-based peer sessions, electricity outages and battery discharge sometimes limit their ability to participate and receive information via online peer sessions.

“Access to internet data can be a barrier because when there is no data, I can’t use Facebook or WhatsApp” AKS-KII-FSW-01.

“When I am talking about WhatsApp or Facebook, the only thing that can hinder me from getting the information might be when I don't have internet data. If I don’t have data, I cannot participate to get the information” AKS-KII-MSM-09.

#### Health system-related factors

Key influencers reiterated that KPs tend to be more worried about the confidentiality of the test results, especially when a facility-based confirmatory test is involved. One major challenge in relaying information on HIVST is the perceived breach of privacy. For example, key influencers have witnessed KPs’ anxieties in communicating positive test results to the key influencers who may want to refer them to health providers at the facilities for a confirmatory test.

“Even if they take the self-testing kit to their house to test themselves and they come out positive, they will not come out to the facility to tell them you are positive” LAS-KII-PE-01.

Key informants interviewed indicated that KPs experience disrespect from providers, especially when the provider is not a community member, ranging from emotional abuse to delays, denial of services, and a bridge of confidentiality and privacy. KPs opined that poor interpersonal communication behaviors of health providers can drive them away from accessing PrEP and HIVST services or even from attending or following up with services.

“Character, it goes a long way, the way the health providers welcome clients, counsel and interact has a lot to do with health-seeking” CRS-KII-FSW-07.

“Sometimes, the health providers talk anyhow to clients. The way you talk to me will make me decide if I want to come back again” CRS-KII-Key influencer-01.

There were also complaints that KPs and their key influencers are not involved in program planning—including initial and evaluation stages. Key influencers think that incorporating KPs and KPIs opinions would enrich the program’s success relating to increasing awareness for the uptake of preventive services.

KP influencers reported limited access to health facilities for referral, coupled with a need for follow-up on the use of the kit, especially in circumstances where the client tests negative and needs to initiate PrEP. In addition, key Influencers complained about poor feedback mechanisms, insisting that KPs do not always return with the feedback of their HIV self-test results, making it difficult to follow up with further PrEP and other HIV services.

“So, the major challenge with self-test kit is that most often when they access this service, they don’t always come back with feedbacks” LAS-KII-Key influencer-01.

#### Socio-cultural factors

KPs expressed fear for social embarrassment and pain associated with usage of ARV for HIV that leads to withdrawal, rejection, and miss opportunities to initiate PrEP among the KPs. There is the concern that their partners and society may inflict emotional and social injuries when found with medicines for PrEP. Some KPs opined that they can trade off life instead of taking PrEP.

“It is just fear – you hear KPs say that they don’t want a situation where they will take it because people believe that it is only those that already have HIV infection that supposes to take ARVs- that is their fear” LAS-KII-HCP-01.

“Because they will be scared of information, some people believe they should die than knowing it (their status) which will cost them so much” LAS-KII-MSM-05.

There is reported discrimination and harassment of KPs by security agencies in Nigeria. That is one of the reasons the KPs especially, MSM, avoid face-to-face community activities where they could learn about PrEP, HIVST, and other prevention approaches. Discrimination sometimes hinders the MSM group in retaining peer discussions and community-related information on their phones due to security personnel interferences to search people's devices.

“When a policeman sees some of us walking like a girl, all you’ll hear is “identify yourself”. If you don't have an ID card, the policeman will go to your phone, abuse and infringe into your right just because of your physical appearance”. LAS-KII-Key influencer-02.

The harassment of the MSM community by government authorities requires privacy if they must receive face-to-face messaging.

“Face to face is good but depends on what information you’re sharing. There is something about our community, and we need assurance that when they gather something else will not happen” LAS-FGD-MSM.

Also, KPs opined that the challenge of peer sessions is that since it's a group-based event, some KPs may not open up, thinking there may be information diffusion to wrong personalities.

Some KPs reported that if PrEP and HIVST messages are presented in languages that they are not conversant with, the objective of the communication is defeated. KPs insisted that the design of PrEP and HIVST messaging must consider clients in rural settings.

#### Socio-economic factors

Some MSM members are unemployed and depend on their families that may not afford the costs or may not want to pay for PrEP and HIVST services. Poor socio-economic status of KPs may limit attention span during online peer sessions. That may lead to an inability of the KPs to invest in internet data and smartphones to participate in online discussions.

“After approaching them, talking to them and showing them how to use it, the price for getting PrEP and HIVST should not be too high so that people can afford it” LAS-KII-Key influencer-01.

“I know that Prep is very expensive. It is not easy to get” LAS-KIIKey influencer-02.

Indirect costs (costs of refreshment and transportation to the venue) associated with participating in peer sessions may hinder the communication of PrEP and HIVST information. Some KPs reported that if the distance to the health facility is very far, they may not want to go there all the time due to indirect costs associated with transportation.

#### Medication-related factors

The indifference of some KPs also limits the acquisition of PrEP and HIVST information, coupled with concerns of why PrEP has to be taken daily and HIVST conducted routinely. These concerns have also led to discontinuations of PrEP use. Among some KPs, there are still doubts about the efficacy of PrEP in protecting an individual, occasioned by advocacy for the combined use of PrEP and condoms for maximum protection. KPs reported that this tends to be cumbersome in practice but would continue using condoms than PrEP.

### Communication needs of KPs for PrEP and HIVST services in Nigeria

#### Receptiveness to future PrEP and HIVST messages

Quantitative data indicated that about 95.5% FSWs and 88.7% MSM indicated they need to receive PrEP messaging in the future. Similarly, 94.2% of FSWs and 86.7% of MSM were willing to receive HIVST messages in the future. Both FGDs and KIIs elicited reasonable and extreme willingness to receive more information on PrEP and HIVST regardless of the typology of KP. KPs are willing to embrace the messages and are eager to disseminate PrEP and HIVST information within and across their networks.

“I need to have more knowledge about it so that I can relate the information to others” AKS-KII-MSM-09.

Most KPs want more information on how to take PrEP, eligibility, clarity on the differences between PrEP and PEP, side effects, and pricing for PrEP.

#### Preferences for communication channels

The preferred channels to receive PrEP and HIVST messages varied across KP typologies. While the FSWs preferred mainly face-to-face, phone calls, and SMS, the MSM group prefer social media-based channels—WhatsApp, Facebook, Instagram, and Twitter. Also, MSMs advocated for other closed social media channels that only admit community members, usually introduced by their peers, including Mangam, Tindre, and Grindre. That is due to their sexual orientation that is stigmatized and criminalized.

“In our community, we are very mobile, almost every community member you see out there is on social media because that's where you meet new people and interact” LAS-KII-MSM-08.

Preference for face-to-face channels was due to the opportunity to ask questions, receive instant feedback, and facilitate understanding. SMS was also not ranked top priority owing to the assumption that the receiver might decide to ignore the messages for a couple of days or may delete them permanently without reading them. SMS might also be subject to misinterpretation.

“Yea, face to face, at least you will understand better” LAS-KII-MSM-03.

“Because it is confidential, so I prefer WhatsApp, nobody has access to my WhatsApp and see what I am chatting” AKS-KII-MSM-09.

There was no consistent pattern and time to receive HIV preventive messages. However, the frequency of receipt of PrEP and HIVST messaging varied among the respondents. The frequency ranged from daily, weekly, and monthly basis.

Brothel-based FSWs would prefer that their chair ladies act as peer educators, enabled with posters, handbills, and other communication materials to improve their work (Table 5).

**Table 5:** Preferred channels of communication for PrEP and HIVST among KPs

	PrEP		HIVST	
	FSW	MSM	FSW	MSM
Face-to-face	67.9	59.2	67.5	47.8
WhatsApp	8.3	17.6	9.3	20.9
Social media	0.3	1.3	5.4	24.6
SMS	20.1	12.4	24.6	20.6
Poster/flier	0.8	1	4.8	1.8
Radio/television	1.9	1.3	6.3	3.5
Other	0.9	7.2	1.7	6.4

For MSMs, the most preferred medium for meeting with a health provider is KP-specific platforms and channels, including the one-stop shops (OSS) that are community-driven and friendly.

“But for me, I think I prefer an NGO that deals with the key population, you don't need to be asked unnecessary questions compared to the general hospitals except in terms of STI” CRS-FGD–MSM.

KPs recommended a disaggregated, targeted communication for maximum coverage and that PrEP and HIVST messaging can be disseminated through churches since almost everyone attends religious gatherings.

### Communication with healthcare providers

There was no definite approach to communicating or consulting with health providers identified in this study. Some KPs prefer to meet their health providers face-to-face. Findings indicated that the face-to-face channel facilitates understanding of sensitive health issues or symptoms. However, there are strong indications for telemedicine among some KPs.

“They can call me on the phone if I need anything, I will tell them to come and meet me here” LAS-KII-FSW-01.

“WhatsApp messages are perfect because it's convenient. Not seeing doctors face to face. Even when I travel to the village, I can still get my message via WhatsApp without issues”. LAS-KII-FSW-03.

While some FSWs prefer to meet older male health providers, some prefer to consult middle-aged female health providers who they perceived would maintain confidentiality for the care they provide.

“I need a mature male provider because they can understand. You can never hear your secret from someone else—a mature person can keep your secret” LAS-KII-FSW-03.

#### Expected future content of the PrEP and HIVST messaging

KPs expressed the need for information on how to take PrEP, eligibility, clarification on differences between PrEP and PEP, clarification on any side effects, pricing, for PrEP, price, efficacy, sales point, dosage, available brands. However, Most KPs preferred to received PrEP and HIVST information in multiple languages: English Language or pidgin English, or local dialect.

## Discussion

This study found intertwining facilitators and barriers to uptake of PrEP and HIVST services and communication needs of KPs for increased access to the HIV prevention services.

Given the low level of awareness about the HIV prevention services (PrEP and HIVST) among the KPs found in this study, strategic planning is necessary to ensure increased access to PrEP and HIVST information to support Nigeria's pursuit to reduce the incidence of new HIV infection. This finding of a low level of awareness was not surprising since Nigeria is yet to roll out a national strategic plan that will enhance awareness and demand generation for PrEP and HIVST services. Cross-cutting factors that may encourage the implementation of sustainable and targeted strategies that are KP-friendly to acquiring PrEP and HIVST messaging were found. The findings suggest that the dissemination of PrEP and HIVST messages will require a targeted and disaggregated audience. Hence, content should consider the preferences of the KP groups for effective and sustainable impacts [20, 21]. Emphasis should be on the specific channels that best attract the attention of the KP groups in dispelling misconceptions, stigma, discrimination, fears, and other barriers that may be contextual to increase the acquisition and use of PrEP and HIVST messages. These findings suggest a well-tailored and implemented national communication strategy with inputs from all key stakeholders, including service providers and end-users [22].

Findings from the key influencers corroborated the thoughts of the KP groups concerning the barriers to acquisition and the use of information to promote PrEP and HIVST behaviour change communication. However, this has been reported in other studies in Nigeria and elsewhere [2, 17, 23]. It was not surprising that stigma, shame, discrimination limit the acquisition and use of HIV prevention messaging in the study areas. That is because individual behaviours are driven by religion and culture, thereby possibly instigating self-stigma and shame. It is necessary to consider these barriers when designing a robust communication strategy that will support increased awareness and access to PrEP and HIVST services [9] while leveraging ongoing cross-cutting plans in eliminating the barriers.

From this study, KPs can effectively network to increase awareness and access to PrEP and HIVST services in Nigeria, as applied to other settings [20, 21]. That is quite encouraging considering the importance of peer influence in producing multiplier effects in behaviours. That will make the peers receptive to the interventions and help reach other peers in their network—especially the hard-to-reach. However, this idea reasonably calls for an opportunity to engage some KPs as champions to increase access to the hidden KPs. Haven established that a total market approach is a vital mechanism to getting the KPs to pay for PrEP and HIVST services [6, 22, 23], our findings on the receptiveness of KPs to receive PrEP and HIVST messages in the future raises high hopes and corroborates with previous studies [24] since the KPs reported initially receiving messages via multiple channels. Multiple channels, including social media, will provide avenues for programmers and key influencers to reinforce messages on future financing plans for PrEP and HIVST when necessary.

This study found that KPs who visit OSS or whose friends visit OSS were more likely to be aware of PrEP than other community members. Therefore, health care providers and key influencers should be ready to provide all-encompassing messaging that will lead to positive behavior change. The contents of the future PrEP and HIVST messaging should reflect the views of the KPs in terms of access points, pricing, efficacy, available brand, among others [8, 15, 25].

## Limitations

This study was formative and conducted only in three states of the federation—Akwa Ibom, C/River, and Lagos and did not represent opinion across the six geopolitical zones. A scale-up of the research across all geopolitical zones and a survey to quantify the prevalence would help understand the dynamics and prioritization of interventions for scaling up PrEP and HIVST services in Nigeria.

During the data collection period, it was difficult to reach the non-brothel-based FSWs to elicit their opinion. Therefore, further research should document the preferences of the non-brothel-based KPs and ensure it reflects in the national communication strategy.

## Conclusion

The policy actors should consider the preferences of the KPs and the key influencers in reducing barriers to communication and increasing the uptake of PrEP and HIVST services and ensure it reflects in a tailored communication strategy. Since multi-linguistics and multi-channels of presentation were enablers to acquiring PrEP and HIVST messages, the communications strategy for HIV prevention should incorporate these recommendations and adapt to context-specific approaches for effective messaging. Understanding that peer influence is essential in propagating PrEP and HIVST messages, program managers should explore avenues that produce multiplier effects in behaviour change in rolling out the tailored communication strategy while maintaining the confidentiality of information and purpose. Parallel programs targeted at increasing uptake of PrEP and HIVST messaging should be aligned to the national communication strategy, for ease of tracking, reporting, and reinforcement. Considering the strong networking effects of KPs in the dissemination of community-related information, programs should continue leveraging the KPs as ambassadors, peer educators, and service providers, especially in linking the hard-to-reach groups within their networks, while considering KP typologies.

## Data Availability

All datasets used and/or analyzed for this study are available on request from the corresponding author. They are currently safely stored at the JSI archive.

## Abbreviations

WHO: World Health Organization
HIV: Human immuno-deficiency virus
AIDS: Acquired immune deficiency syndrome
KPs: Key populations
MSM: Men who have sex with men
FSWs: Female sex workers
ART: Antiretroviral therapy
PrEP: Pre-exposure prophylaxis
HIVST: HIV self-testing
ODK: Open data kit
SPSS: Statistical Package for Social Sciences
NHREC: National Health Research Ethics Committee
JSI: John Snow Incorporated
